# Genomic Insights into *Paucibacter aquatile* DH15, a Cyanobactericidal Bacterium, and Comparative Genomics of the Genus *Paucibacter*

**DOI:** 10.4014/jmb.2307.07008

**Published:** 2023-09-28

**Authors:** Ve Van Le, So-Ra Ko, Hee-Mock Oh, Chi-Yong Ahn

**Affiliations:** 1 Cell Factory Research Centre, Korea Research Institute of Bioscience & Biotechnology, Daejeon 34141, Republic of Korea; 2 Department of Environmental Biotechnology, KRIBB School of Biotechnology, University of Science and Technology, Daejeon 34113, Republic of Korea

**Keywords:** Cyanobactericidal bacteria, *Paucibacter*, cyanobacteria, *Microcystis*

## Abstract

*Microcystis* blooms threaten ecosystem function and cause substantial economic losses. Microorganism-based methods, mainly using cyanobactericidal bacteria, are considered one of the most ecologically sound methods to control *Microcystis* blooms. This study focused on gaining genomic insights into *Paucibacter aquatile* DH15 that exhibited excellent cyanobactericidal effects against *Microcystis*. Additionally, a pan-genome analysis of the genus *Paucibacter* was conducted to enhance our understanding of the ecophysiological significance of this genus. Based on phylogenomic analyses, strain DH15 was classified as a member of the species *Paucibacter aquatile*. The genome analysis supported that strain DH15 can effectively destroy *Microcystis*, possibly due to the specific genes involved in the flagellar synthesis, cell wall degradation, and the production of cyanobactericidal compounds. The pan-genome analysis revealed the diversity and adaptability of the genus *Paucibacter*, highlighting its potential to absorb external genetic elements. *Paucibacter* species were anticipated to play a vital role in the ecosystem by potentially providing essential nutrients, such as vitamins B_7_, B_12_, and heme, to auxotrophic microbial groups. Overall, our findings contribute to understanding the molecular mechanisms underlying the action of cyanobactericidal bacteria against *Microcystis* and shed light on the ecological significance of the genus *Paucibacter*.

## Introduction

Cyanobacteria are crucial primary producers and indispensable in the food web of aquatic environments [1]. These organisms, however, release multiple cyanotoxins, such as microcystins, cylindrospermopsins, and anatoxins [2]. Under favorable conditions, the proliferation of cyanobacteria leads to dense blooms, known as cyanobacterial blooms, which negatively affect water quality and public health [3]. *Microcystis* is one of the most prevalent bloom-forming cyanobacteria [4]. Owing to global warming, *Microcystis* blooms are expected to become more widespread [5]. Therefore, finding effective strategies for controlling cyanobacterial blooms is an important research topic.

There is much attention on microorganism-based methods, mainly using cyanobactericidal bacteria, to control cyanobacterial blooms because of their eco-friendliness [6]. Several bacteria, such as *Aeromonas* [7], *Enterobacter* [8], and *Brevibacillus* [9], have been reported to exhibit cyanobactericidal activity against cyanobacteria. They have been isolated from various ecological niches, such as soil [10], sediment [11], and cyanobacterial blooms [12]. Cyanobactericidal bacteria can directly [13] and/or indirectly [12, 14] inhibit the growth of cyanobacteria. Based on our previous studies, *Paucibacter aquatile* DH15 exhibited a significant potential for effective control of cyanobacterial blooms [12, 15, 16]. The cyanobactericidal activity of DH15 can be mediated by a combination of physical attachment and indirect attack [12]. The interactions between cyanobactericidal bacteria and cyanobacteria in aquatic ecosystems are complicated and diverse [17]. Despite ongoing research, our current understanding of the molecular mechanism behind cyanobactericidal action remains inadequate. Although the mechanism may be even more intricate than previously thought, analyzing the genome of cyanobactericidal bacteria can provide valuable insights into their mechanisms of action against cyanobacteria. However, few studies have investigated the genomic features of cyanobactericidal bacteria [18].

The genus *Paucibacter* belonging to the family *Comamonadaceae* was first described by Rapala *et al*. [19], with *Paucibacter toxinivorans* as the type species. *Paucibacter* is rod-shaped, Gram-negative, weakly catalase-positive [19] and currently consists of three species: *P. toxinivorans* [19], *P. oligotrophus* [20], and *P. aquatile* [21]. These bacteria were isolated from sediment [19] and fresh water [20, 21]. Although high-quality whole genome sequences of 13 *Paucibacter* strains have been published, in-depth research has not been done on genomic traits and ecological roles of the genus *Paucibacter* [22, 23].

Therefore, this study aimed to (1) report the complete genome sequence of strain DH15; (2) determine the taxonomic position of strain DH15; (3) suggest the cyanobactericidal mechanism of DH15 against *Microcystis* through genomic analysis; and finally, (4) explore the ecological functions of the genus *Paucibacter* through pan-genome analysis. To our knowledge, this is the first study to investigate the comparative genomics of the genus *Paucibacter*.

## Materials and Methods

### Bacterial Strain

Strain DH15 was isolated from a freshwater sample collected from Daechung Reservoir (36° 25' 56'' N, 127° 33'02'' E), Korea [12]. The strain was maintained, preserved as described previously [24], and deposited in Korean Collection for Type Cultures (KCTC) with the accession number KCTC 18831P.

### Co-Culture of *Microcystis aeruginosa* and DH15

*M. aeruginosa* strain KW was inoculated into in BG-11 medium (Sigma-Aldrich, USA) and incubated under continuous illumination (80 μmol photons/m^2^/s) at 25°C. Strain DH15 was co-cultured with *M. aeruginosa* strain KW as mentioned previously [12]. Briefly, strain DH15 grown on R2A (Difco, USA) plates for 2 days at 25°C was harvested and resuspended into BG11 medium. *M. aeruginosa* culture (2 × 10^6^ cells/ml) was inoculated with DH15 cultures (2.1 × 10^6^ colony forming units/ml) at a concentration of 1% (v/v). *M. aeruginosa* cells were observed under a transmission electron microscopy according to Le *et al*. [12].

### DNA Extraction and Phylogenetic Analysis

After strain DH15 was grown on R2A medium at 25°C for 2 days, total genomic DNA was extracted using a FastDNA Spin DNA extraction (MP Biomedicals, USA) following the manufacturer's instructions. The 16S rRNA gene of strain DH15 was amplified and sequenced using the universal primer set 27F (5'-AGAGTTTGATCATGG CTCAG-3') and 1492R (5'-TACGGYTACCTTGTTACGACTT-3') [25]. Phylogenetic trees based on 16S rRNA gene sequences were constructed with the MEGA 11 software [26], using the maximum-likelihood (ML), neighbor-joining (NJ), and minimum evolution (ME) algorithms with 1000 bootstrap iterations.

### Genome Sequencing

The DH15 genome was sequenced using Pacific Biosciences (PacBio) SequelII system and Illumina platform (Macrogen, Republic of Korea). PacBio HiFi (High Fidelity) reads were assembled using SMRT link v11. After assembly, Illumina reads were used for accurate genome sequencing with Pilon v1.21 [27]. The depth of coverage data was generated by mapping the HiFi reads against assembled contigs with minimap2 [28]. Benchmarking Universal Single-Copy Orthologs (BUSCO, v3.0) was employed to assess the completeness of the genome assembly [29]. After the genome was assembled, the gene prediction and functional annotation were performed using the NCBI Prokaryotic Genome Annotation Pipeline (PGAP), Rapid Annotation Subsystem Technology (RAST) [30], and PATRIC server [31]. The clusters of orthologous group (COG) functional categories were annotated using eggNOG-mapper 2.1.9 [32]. The genome sequence of strain DH15 is available at DDBJ/EMBL/GenBank under the accession number CP124551. The putative secondary metabolite biosynthetic gene clusters were identified by the antiSMASH server, with detection strictness set as “strict” [33]. Genes encoding for the Carbohydrate-Active enZYme (CAZyme) were identified and annotated using dbCAN2 meta server (http://bcb.unl.edu/dbCAN2/) [34].

### Phylogenomic Analysis

Average nucleotide identity (ANI) and digital DNA–DNA hybridization (dDDH) values between strain DH15 and other *Paucibacter* strains were calculated using the anvi’o program [35] and Genome-to-Genome Distance Calculator formula-2 [36], respectively. The phylogenomic tree was constructed by Type (Strain) Genome Server (TYGS) to identify taxonomic position of strain DH15 [37].

### Pan-Genome Analysis

To investigate the diversity of the genus *Paucibacter*, genome data of *Paucibacter* strains were retrieved from the NCBI database (Table 1). The genome sequences of *Paucibacter* strains were selected based on their quality, with a minimum genome coverage of 80× and the presence of publicly available information about their isolation source. Their genomic features, such as genome coverage, genome size, number of genes and proteins, and GC% were obtained from the NCBI database. Pan-genome was built using Roary with a minimum percentage identity of 80%for BLASTp [38]. All genes were identified as core, shell, and cloud genes based on their presence among the genomes analyzed. When specific genes were present in all the genomes and more than 15% of the genomes, they were designated as core and shell genes, respectively. Core/pan-genome plot was visualized using GraphPad Prism software v.9.0.2 (GraphPad Software, Inc., USA). The pan-genome figures were visualized using the anvi’o platform [35]. A flower plot showing the core genome and strain-specific genes was generated using EVenn (http://www.ehbio.com/test/venn). Metabolic pathways were reconstructed using BlastKOALA of KEGG [39].

## Results and Discussion

### Identification and Genome Feature of Strain DH15

Initially, we identified the taxonomic position of the strain using its 16S rRNA gene sequence. Comparison of the 16S rRNA gene sequences showed that strain DH15 was most closely related to *P. aquatile* CR182^T^ (99.64%). All the phylogenetic trees based on 16S rRNA gene sequences indicated that the strain forms a robust cluster with *P. aquatile* CR182^T^ with a high bootstrap support value of 100% (Figs. S1-S3).

**Fig. 1:**
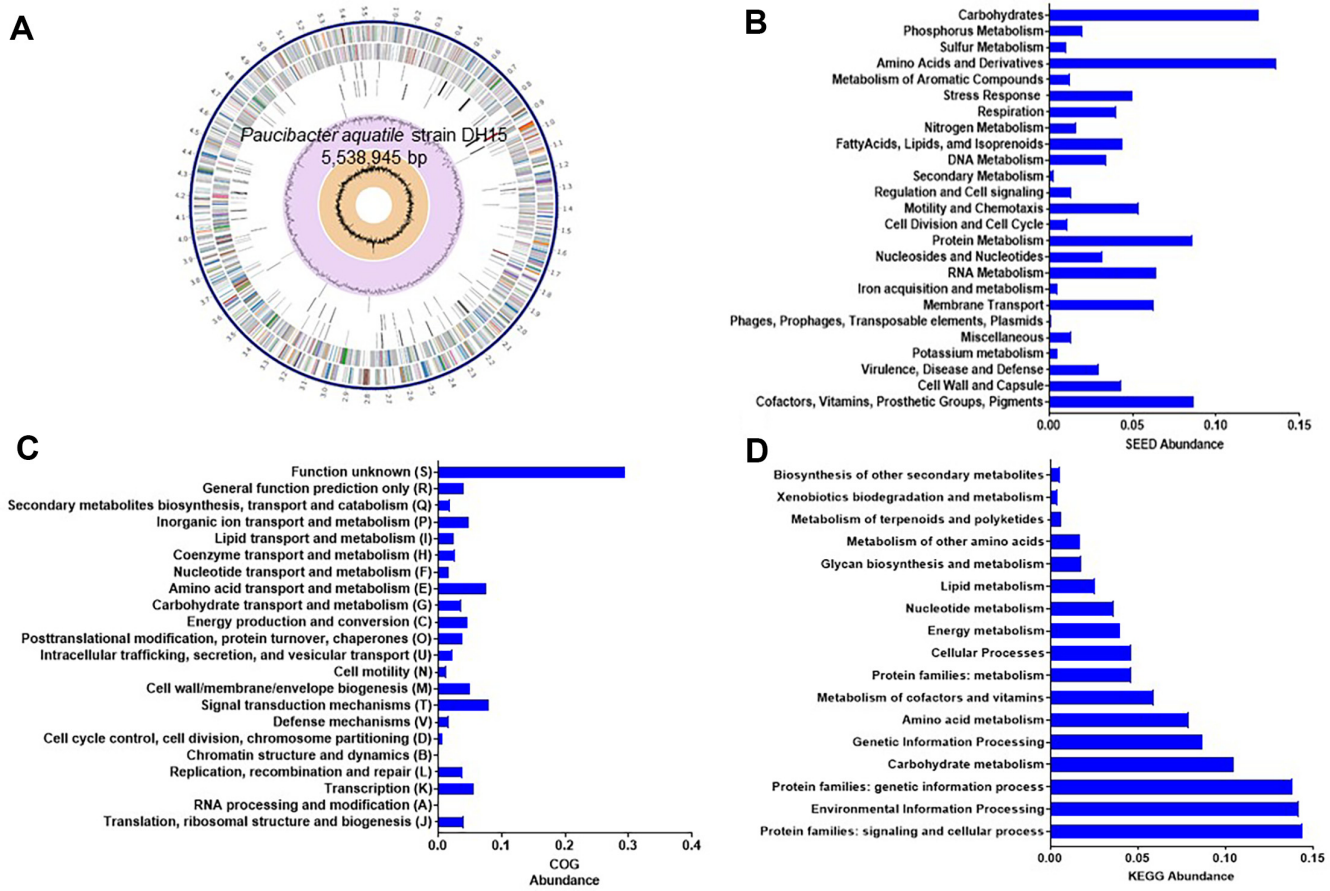
Genome feature of strain DH15. (**A**) Circular view of the genome of strain DH15 created by PATRIC. From outside to center, the contigs, CDS on the forward strand, CDS on the reverse strand, RNA genes, CDS with homology to known antimicrobial resistance genes, CDS with homology to known virulence factors, GC content, and GC skew. (**B**-**D**) SEED subsystem categorization (**B**), KEGG category (**C**), and COG abundance (**D**) of strain DH15.

The genome sequence of strain DH15 was assembled into a single circular chromosome consisting of 5,538,945 bases, with a GC content of 66.4% (Fig. 1A). Annotation of the genome using NCBI-PGAP identified 4,604 coding sequences, 9 rRNAs, 62 tRNAs, and 5 ncRNA. Gene prediction and annotation by the RAST server and COG analysis revealed the high relative abundance of genes associated with crucial cellular functions. These functions encompassed various metabolic processes such as the metabolism of carbohydrates, amino acids and their derivatives, proteins, cofactors, vitamins, prosthetic groups, pigments, and membrane transport (Fig. 1B, 1C). In the KEGG analysis, a large portion of the subsystem was dedicated to processing environmental information (Fig. 1D).

**Fig. 2:**
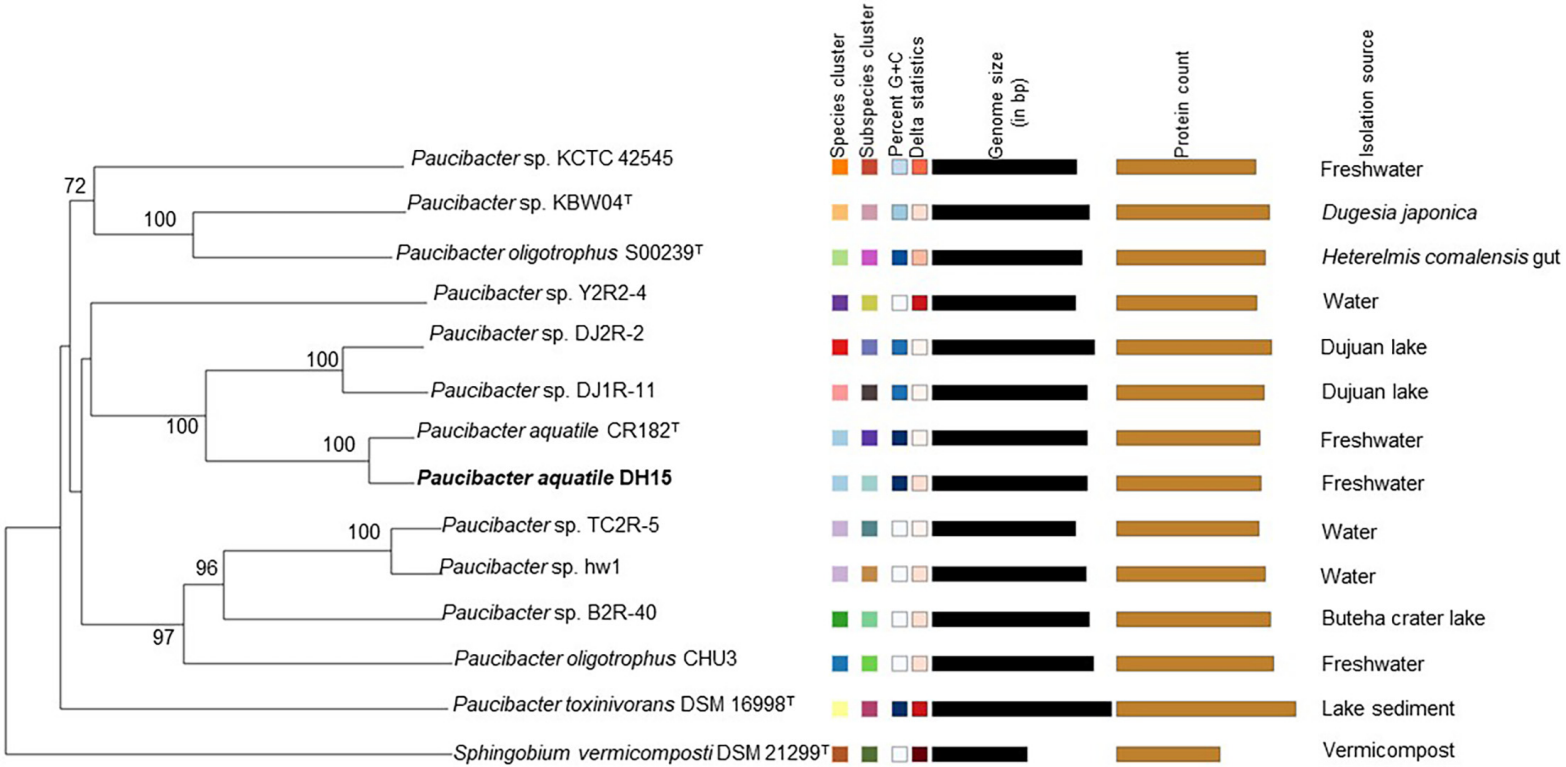
Phylogenomic tree constructed using Type (Strain) Genome Server depicting the position of strain DH15 among the strains of the genus *Paucibacter*. The branch lengths are scaled in terms of GBDP distance formula d5. The numbers at the nodes are GBDP pseudo-bootstrap support values.

Since 16S rRNA has limited discriminatory power, genome-level analysis was further used to ascertain the taxonomic status of strain DH15. ANI and dDDH are major genomic metrics for species discrimination [40], with threshold values of 95% (ANI) [41] and 70% (dDDH) [42] commonly used for species delineation. Therefore, we calculated ANI and DDH values of strain DH15 with its closely related type strains. DH15 shared the highest ANI and DDH values of 97.11 and 75.5%, respectively, with *P. aquatile* CR182^T^ (Fig. S4). Accordingly, strain DH15 belonged to the species *P. aquatile*. This conclusion was further supported by the phylogenomic tree analysis, which showed that strain DH15 clustered together with *P. aquatile* CR182^T^ (Fig. 2). Notably, strains KBW04, CHU3, DJ1R-11, DJ2R-2, TC2R-5, Y2R2-4, hw1, and KCTC42545 exhibited ANI and dDDH values below the thresholds established for species delineation when compared to other *Paucibacter* species, suggesting their novel genospecies status in the genus *Paucibacter* (Fig. S4). *Paucibacter* species were isolated from freshwater, lake, sediment, *Heterelmis comalensis* gut, and *Dugesia japonica* (Table 1). No clear relationship between evolutionary relationship and isolation source was observed (Fig. 2).

### Genomic Insight for the Cyanobactericidal Activity of Strain DH15

Cyanobactericidal bacteria can destroy *Microcystis* by physical attachment [13] and/or by secretion of cyanobactericidal compounds [10] and hydrolytic exo-enzymes [43]. In our previous study, cyanobactericidal activity of DH15 was mediated by both physical attachment and secretion of cyanobactericidal compounds [12]. The flagellar motility of microorganisms allows them to compete effectively with other microbes [44]. Flagellated bacteria have the ability of chemotaxis, which is directed toward prey microalgae [45]. *P. aquatile* has been reported to be motile by a single polar flagellum [21]. The EggNOG-mapper showed that strain DH15 contained genes associated with flagellar motility, such as *fliR*, *fliQ*, *flgI*, and *flgH*, suggesting that this strain utilizes flagellar motility for chemotactic movement to *Microcystis* (Table 2). This motility of strain DH15 is likely to enhance its ability to approach and interact with *Microcystis* cells, potentially leading to the disruption or control of *Microcystis* populations.

Amino-acid derivatives are considered one of the most frequently identified cyanocides [45]. Phenylalanine inhibits microcystin production and damages the photosynthetic system in *Microcystis*, leading to cell death [46]. Lysine may impair peptidoglycan synthesis, causing irreversible harm to the photosynthetic system and membrane integrity [47]. After a three-day treatment, the growth of *M. aeruginosa* was significantly inhibited (< 90%) by L-lysine at 5.0 mg l^−1^ [48]. Based on BlastKOALA [39], a complete pathway for phenylalanine (M00024) and lysine (M00016) synthesis was constructed in the genome of strain DH15, supporting its algicidal activity against *Microcystis*.

**Fig. 3:**
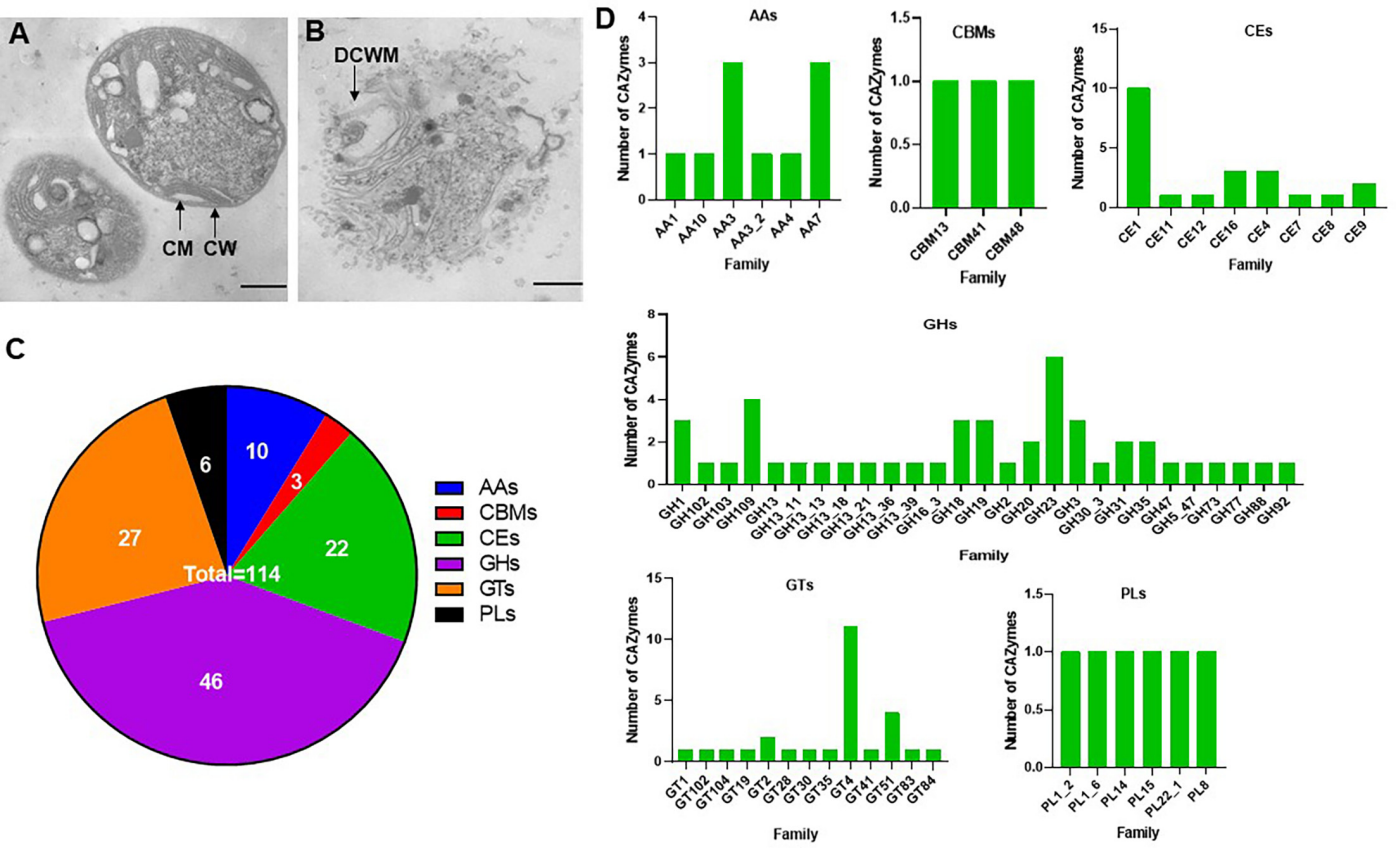
Genomic insight for the cyanobactericidal activity of strain DH15. Morphology of *M. aeruginosa* strain KW (**A**) and its morphological response to strain DH15 after 12 h of exposure (**B**). CM, cell membrane; CW, cell wall; DCWM, damaged cell wall and membrane. Scale bar: 1 μm. (**C**) Carbohydrate-active enzymes (CAZymes) annotated in strain DH15 using the CAZymes Analysis Toolkit. (**D**) The number of CAZymes in each family. CBMs, Carbohydrate-Binding Modules; CEs, carbohydrate esterases; GTs, glycosyltransferases; AAs, auxiliary activities; PLs, polysaccharide lyases; and GHs, glycoside hydrolases.

Cell wall constituents of *Microcystis* include carotenoids, two major peptidoglycan-associated proteins, and lipopolysaccharide [49]. CAZymes are key enzymes involved in the deconstruction and modification of complex carbohydrates [34]. Cyanobactericidal bacteria use CAZymes as a vital virulence factor to attack cyanobacteria [50]. For example, *Tramates versicolor* F21a secretes glycoside hydrolase, coenzyme, carbohydrate esterase, and polysaccharide lyases, which degrade lipopolysaccharide, peptidoglycan, and alginate in cyanobacterial cells, leading to death [50]. During the algae-lysing process of *Brevibacillus laterosporus*, the expression of glycoside hydrolase family 18 (GH18) encoding chitinase was increased [9]. Transmission electron microscopic observation revealed that strain DH15 lysed the cell wall and membrane of *Microcystis* during the interaction (Fig. 3A-3B). This result suggested that DH15 may produce polysaccharide-degradative enzymes. Strain DH15 genome contained lots of diverse CAZymes. A total of 114 CAZyme genes were identified in the genome of strain DH15, including those encoding 46 glycoside hydrolases (GHs), 27 glycosyltransferases (GTs), 22 carbohydrate esterases (CEs), 10 auxiliary activities (AAs), 6 polysaccharide lyases (PLs), and three proteins with carbohydrate-binding modules (CBMs) (Fig. 3C-3D). GHs are enzymes hydrolyzing the glycosidic bonds between two or more carbohydrates or between a carbohydrate and a non-carbohydrate moiety [51]. This family was most abundant among CAZyme families in strain DH15. Strain DH15 can hydrolyze peptidoglycan with peptidoglycan lyase (GH23) [52]. GTs (EC 2.4.x.y) catalyze the formation of glycosidic linkages to form glycosides, using sugar donors containing a nucleoside phosphate or a lipid phosphate leaving group [53]. Among the identified GTs, the GT4 family (11 genes) and GT51 family (4 genes) were present in a larger proportion (Fig. 3D). CEs catalyze the de-O or de-N-acylation of substituted saccharides to remove esters from substituted saccharides [54]. CBMs are noncatalytic modules promoting the association of all other CAZymes with the substrates [55]. Taken together, these CAZymes in strain DH15 are likely to contribute to polysaccharide degradation and resource utilization within *Microcystis* colonies for its own growth and survival.

Prodigiosin has been reported to have many biological activities, such as antibacterial, anticancer, and immunomodulatory properties [56]. This compound exhibits cyanobactericidal activity against *Microcystis* by inducing oxidative stress and damaging the photosynthetic system [57]. The prodigiosin biosynthesis gene cluster predicted in the genome of strain DH15 using antiSMASH supported its algicidal activity against *Microcystis* (Table 3). By inhibiting the growth and survival of *Microcystis*, strain DH15 may mitigate *Microcystis* bloom, promoting the ecological balance of aquatic ecosystems.

Pradimicin A is a nonpeptidic benzonaphtacenequinone antibiotic with antifungal properties. It has also shown a promise as a potential therapeutic compound for human immunodeficiency virus (HIV) therapy [58]. Longicatenamide A has been found to possess antimicrobial activity against *Bacillus subtilis* [59]. Chitinase can lyse the cell wall of fungi and has recently gained attention as an alternative and safe antifungal agent [60, 61]. The presence of biosynthesis gene clusters for pradimicin A, longicatenamide A, chitinases (GH19 and GH18) in the genome of strain DH15 suggests that this strain possesses not only cyanobactericidal activity but also antifungal and antimicrobial properties (Table 3 and Fig. 3).

### Pan- and Core-Genome Analysis of *Paucibacter* Strains

**Fig. 4:**
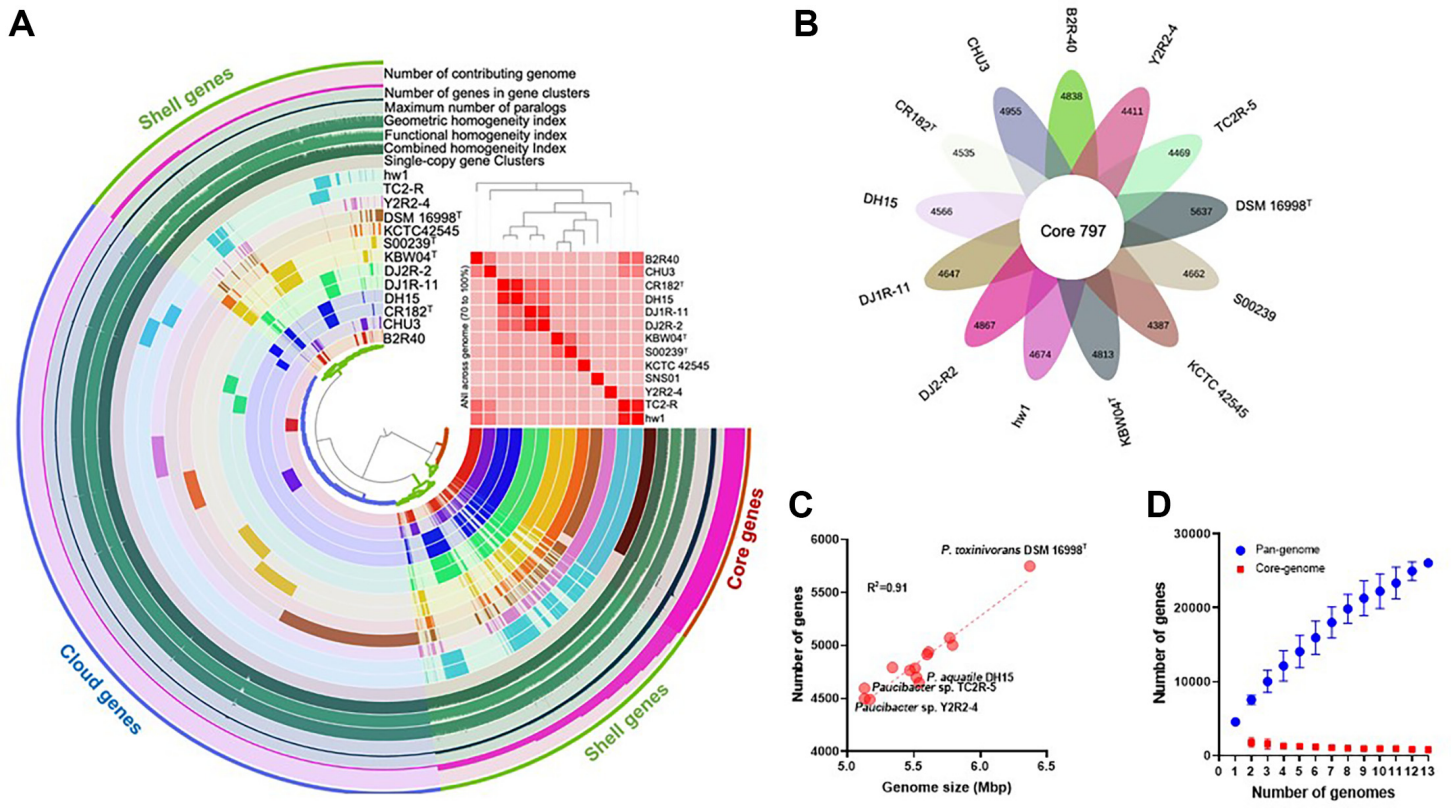
Pan- and core-genome analysis of *Paucibacter* strains. (**A**) The pan-genome analysis of *Paucibacter* using anvi’o workflow. (**B**) The data points within 13 layers indicate the presence of a gene cluster in a given genome. Flower diagram showing the core and unique genes of the *Paucibacter* strains calculated by Roary. (**C**) Correlation between the genome size and the number of genes of the genus *Paucibacter*. (**D**) Core/pan-genome plot of *Paucibacter* species using Roary.

The pan-genome analysis is a robust method to investigate the diversity of bacterial species and may unveil an evolutionary history of the genus [62]. In this study, for the first time, the pan-genome of the genus *Paucibacter* was analyzed. The proportion of core, shell, and cloud genes was estimated to be 10.14, 36.23, and 53.63%, respectively (Fig. 4A). Specifically, we identified 797 core genes (Fig. 4B). The genome size of *Paucibacter* species ranged from 5.13 for *Paucibacter* sp. TC2R-5 and *Paucibacter* sp. Y2R2-4 to 6.37 Mb for *P. toxinivorans* DSM 16998T (Fig. 4C) with an average genome size of 5.54 Mb. The %G+C mean content of the genus *Paucibacter* was 65.3 ± 1.5%. Since the addition of newly sequenced *Paucibacter* strains increased novel genes, the pan-genome of *Paucibacter* species belongs to the open pan-genome category (Fig. 4D) [63]. Therefore, *Paucibacter* species may incorporate external genetic material and expand genetic diversity through recombination and mutation [63]. This ability allows them to adapt to diverse habitats by acquiring accessory genes and maintaining a flexible gene pool [63]. However, the possibility still remains that adding more genomes of newly found species of *Paucibacter* could reveal the feature of closed pan-genome.

**Fig. 5:**
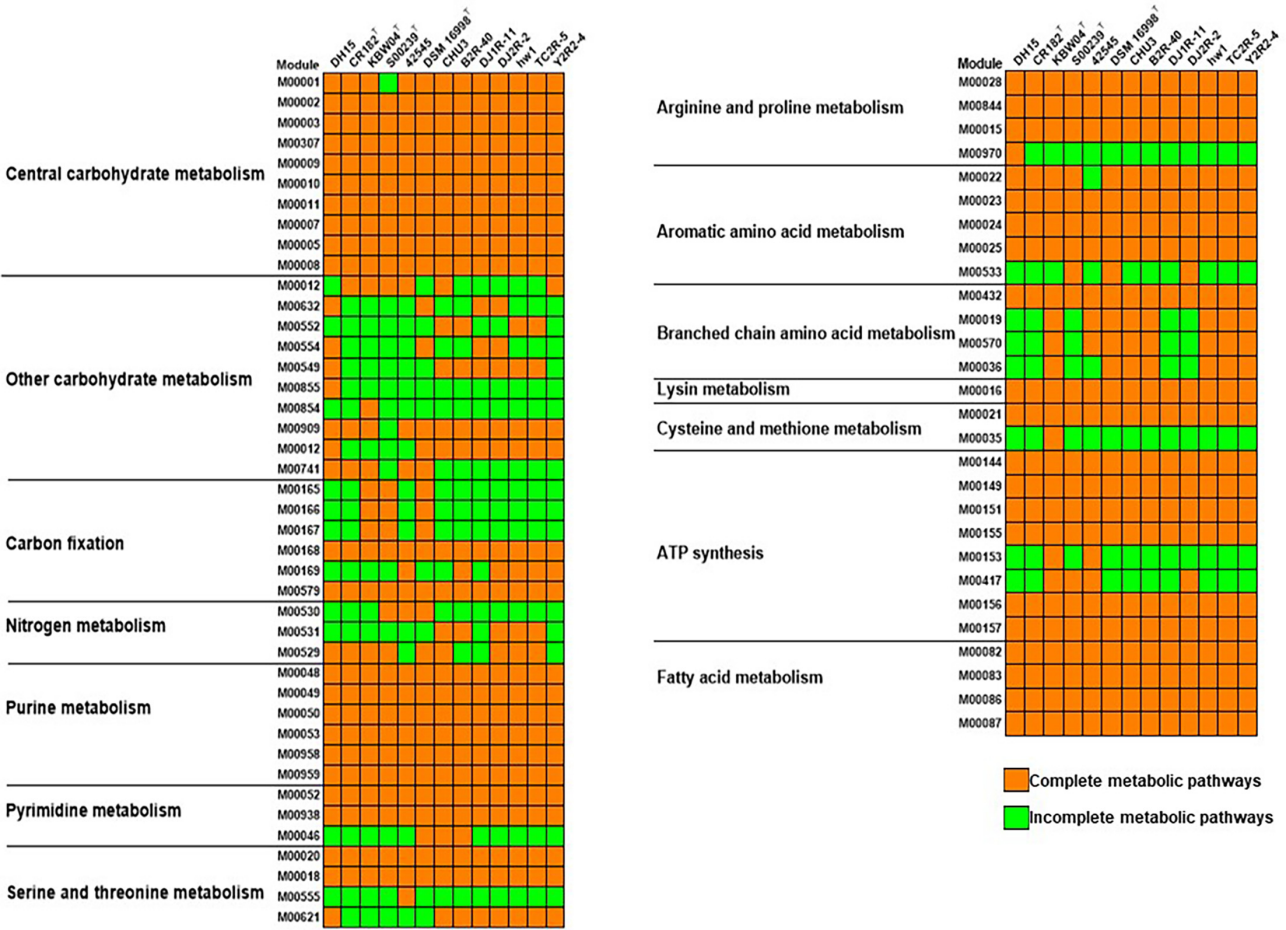
Heatmaps of complete and incomplete metabolic pathways in the genomes of *Paucibacter* strains predicted by BlastKOALA.

KEGG analysis showed that *Paucibacter* species exhibited a generally similar distribution pattern of critical central metabolic pathways such as central carbohydrate metabolism, purine metabolism, ATP synthesis, and fatty acid metabolism (Fig. 5 and Table S1). For example, all strains contained complete pathways of M00002, M00003, and M00307, which are involved in glycolysis, gluconeogenesis, and pyruvate oxidation, respectively. The annotation of numerous genes encoding RNAs, including rRNA and tRNA, suggests that they can promptly adapt to changing environmental conditions and exhibit rapid growth when nutrients are abundant (Table 1) [64].

### Potential Ecological Roles of *Paucibacter* Species

**Fig. 6:**
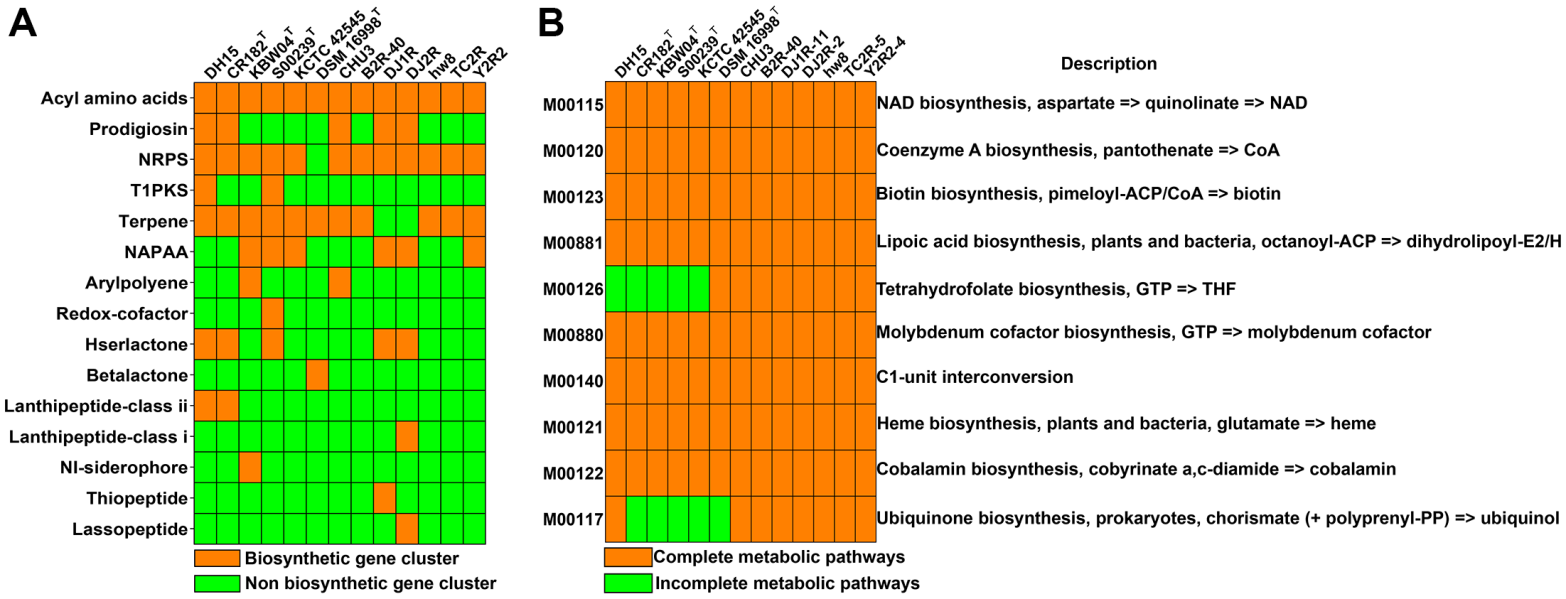
Potential ecological roles of *Paucibacter* species. (**A**) Predicted secondary metabolite biosynthetic gene clusters of *Paucibacter* strains. (**B**) Cofactor and vitamin metabolism of *Paucibacter* strains. NRPS, Non-ribosomal peptide synthetase; T1PKS, Type I PKS (Polyketide synthase); NAPAA, Non-alpha poly-amino acids; NI-siderophore, NRPS-independent IucA/ IucC-like siderophore; NAD, Nicotinamide adenine dinucleotide; CoA, Coenzyme A; GTP, Guanosine triphosphate; and THF, Tetrahydrofolate.

Secondary metabolites produced by microbes play critical roles in their communication with neighbors for defense, cooperation, co-evolution, and competition [65]. Also, they are elemental sources for discovering novel antimicrobial and bioactive compounds [33]. The next-generation sequencing technologies can give us insights into the metabolic potential of bacteria as producers of secondary metabolites [66]. The antiSMASH analysis revealed considerable variability in the secondary metabolite profiles among *Paucibacter* strains (Fig. 6A), highlighting their diverse defense mechanisms against other microorganisms. The genomes of all *Paucibacter* strains contained biosynthetic gene clusters for acyl amino acids, and most strains possessed gene clusters for non-ribosomal peptide synthetase (NRPS) and terpene production (Fig. 6A). These secondary metabolites seem to contribute to the competitive advantage and defense capabilities of *Paucibacter* species against competitors and predators in their ecological niche [67-71]. In addition to strain DH15, the genomes of strains CR182^T^, CHU3, DJ1R, and DJ2R were found to have biosynthetic gene clusters for producing prodigiosin. This finding indicates that these strains could exhibit cyanobactericidal activity [57] and play an essential role in regulating cyanobacterial blooms.

B vitamins produced by microorganisms are crucial cofactors for all living organisms [72]. The supply of vitamins in bacteria may be achieved either by *de novo* synthesis or import from exogenous sources [73]. Phytoplankton acquire vitamin B and nutrients through mutualistic interactions with bacteria [74]. All *Paucibacter* species had the complete pathway of biotin (vitamin B_7_) and cobalamin (vitamin B_12_) biosynthesis (Fig. 6B). Vitamin B_7_ is required in amino acid metabolism, fatty acid biosynthesis [75], and DNA damage prevention [76]. Vitamin B_12_, known as cyanocobalamin, has been reported to reduce DNA damage [77], control DNA biosynthesis [78], and alter the composition of microbial communities [79]. Heme, a metal prosthetic group of several proteins, relates to diverse metabolic and respiratory processes across all organisms [80]. Since many organisms cannot produce heme, they must assimilate exogenous heme from the environment [80]. The production of heme, vitamins B_7_, and B_12_ by all *Paucibacter* species appears crucial for their mutualistic interactions with other microbial groups that rely on external sources of these vitamins and heme.

In summary, the present study provides insights into the genome characteristics of strain DH15, highlighting its cyanobactericidal activity against *Microcystis* through flagellar movement, cell wall degradation, and the production of cyanobactericidal compounds. Furthermore, the genome analysis revealed the potential for antifungal activity in strain DH15, although further investigations are necessary for confirmation. The ecological role of *Paucibacter* species potentially involves supplying vital nutrients like vitamins B_7_, B_12_, and heme to neighbors that lack the *de novo* biosynthetic pathway of these nutrients. The discovery of an open pan-genome in *Paucibacter* suggests that this genus is highly good at incorporating external genetic elements from the surrounding environment. Such genomic plasticity is likely a key factor contributing to the adaptability and overall success of *Paucibacter* species.

## Supplemental Materials

Supplementary data for this paper are available on-line only at http://jmb.or.kr.



## Figures and Tables

**Table 1:** Genome information of strain DH15 and other *Paucibacter* strains used in this study.

Strain	Isolation source	GenBank Accession No.	Genome coverage (×)	Size (MB)	G+C (%)	Gene	Protein coding genes	RNA genes
*Paucibacter aquatile* DH15	Fresh water	CP124551	425.5	5.54	66.4	4,680	4,569	76
*Paucibacter aquatile* CR182^T^	Fresh water	POSP00000000	230.9	5.52	66.3	4,700	4,558	106
*Paucibacter* sp. KBW04^T^	*Dugesia japonica*	QAJN00000000	86.4	5.60	63.7	4,915	4,822	63
*Paucibacter oligotrophus* S00239^T^	*Heterelmis comalensis* gut	JACHLP000000000	281.0	5.34	65.8	4,793	4,665	68
*Paucibacter* sp. KCTC 42545	Fresh water	CP013692	114.0	5.17	63.2	4,491	4,352	102
*Paucibacter toxinivorans* DSM 16998^T^	Lake sediment	SNXS00000000	169.0	6.37	66.4	5,748	5,619	62
*Paucibacter oligotrophus* CHU3	Fresh water	JAJIRN000000000	192.0	5.77	62.0	5,072	4,944	58
*Paucibacter* sp. B2R-40	Buteha crater lake	JAJIRP000000000	214.0	5.61	61.67	4,939	4,813	59
*Paucibacter* sp. DJ1R-11	Dujuan lake	JAJIRR000000000	223.0	5.51	65.34	4,787	4,661	69
*Paucibacter* sp. DJ2R-2	Dujuan lake	JAJIRT000000000	311.0	5.79	65.19	5,004	4,869	63
*Paucibacter* sp. hw1	Water	JAQQXT000000000	151.0	5.47	61.81	4,765	4,665	60
*Paucibacter* sp. TC2R-5	Water	JAJIRQ000000000	270	5.13	62.06	4,596	4,469	63
*Paucibacter* sp. Y2R2-4	Water	JAJIRO000000000	252	5.13	62.00	4,498	4,384	48

**Table 2:** Motility-related genes in *Paucibacter aquatile* DH15 annotated using EggNOG-mapper.

Locus Tag	Start	End	Gene	Function
DH15_1_00342	386269	387531	*yscU*	Yop proteins translocation protein U
DH15_1_01491	1738799	1739563	*fliR*	Flagellar biosynthetic protein FliR
DH15_1_01492	1739563	1739832	*fliQ*	Flagellar biosynthetic protein FliQ
DH15_1_01493	1739853	1740617	*fliP_2*	Flagellar biosynthetic protein FliP
DH15_1_01495	1741122	1741559	*fliN*	Flagellar motor switch protein FliN
DH15_1_01496	1741549	1742553	*fliM*	Flagellar motor switch protein FliM
DH15_1_01502	1747749	1748744	*fliG*	Flagellar motor switch protein FliG
DH15_1_01503	1748795	1750477	*fliF*	Flagellar M-ring protein
DH15_1_01504	1750738	1751061	*fliE*	Flagellar hook-basal body complex protein FliE
DH15_1_01508	1752893	1753324	*fliS*	Flagellar secretion chaperone FliS
DH15_1_01509	1753583	1755013	*fliD*	Flagellar hook-associated protein 2
DH15_1_01510	1755195	1756040	*hag_1*	Flagellin
DH15_1_01511	1756392	1757237	*hag_2*	Flagellin
DH15_1_01512	1757634	1758473	*hag_3*	Flagellin
DH15_1_01521	1765976	1766833	*motA*	Motility protein A
DH15_1_01522	1767038	1767997	*motB*	Motility protein B
DH15_1_01525	1769918	1771102	*flhB*	Flagellar biosynthetic protein FlhB
DH15_1_01526	1771099	1773198	*flhA_2*	Flagellar biosynthesis protein FlhA
DH15_1_01527	1773195	1774895	*ftsY_1*	Signal recognition particle receptor FtsY
DH15_1_01541	1794295	1795428	*flgI*	Flagellar P-ring protein
DH15_1_01542	1795474	1796184	*flgH*	Flagellar L-ring protein
DH15_1_01543	1796199	1796981	*flgG*	Flagellar basal-body rod protein FlgG
DH15_1_01544	1797010	1797735	*flgF*	Flagellar basal-body rod protein FlgF
DH15_1_01545	1797791	1799101	*flgE*	Flagellar hook protein FlgE
DH15_1_01547	1799823	1800239	*flgC*	Flagellar basal-body rod protein FlgC
DH15_1_01548	1800279	1800692	*flgB*	Flagellar basal body rod protein FlgB

**Table 3:** Gene clusters of antimicrobial metabolites identified from *Paucibacter aquatile* DH15 using antiSMASH.

Predicted metabolites	Gene cluster type	Sequence size (bp)	Antimicrobial action
Longicatenamide A, B, C, D	NRPS, T1PKS	67,527	Antimicrobial
-	lanthipeptide-class-ii	22,988	-
-	terpene	21,808	-
-	NRPS	44,224	-
Prodigiosin	hserlactone, prodigiosin	44,698	Antibacterial, antifungal, antiprotozoal, antimalarial
-	acyl amino acids	59,774	-
-	acyl amino acids	60,052	-
Pradimicin-A	acyl amino acids	60,834	Antifungal
