# Supplementary material for: Genomic Insights into Paucibacter aquatile DH15, a Cyanobactericidal Bacterium, and Comparative Genomics of the Genus Paucibacter
Source: J Microbiol Biotechnol. 2023 Sep 28;33(12):1615–24. doi: 10.4014/jmb.2307.07008 (PMC10772561; doi:10.4014/jmb.2307.07008)
Supplement: Supplementary file 1 [file jmb-33-12-1615-supple.pdf]

J. Microbiol. Biotechnol.
https://doi.org/10.4014/jmb.2307.07008

Supplementary Table and Figures

Genomic insights into *Paucibacter aquatile* DH15, a cyanobactericidal bacterium, and comparative genomics of the genus *Paucibacter*

Ve Van Le[1], So-Ra Ko[1], Hee-Mock Oh[1,2], Chi-Yong Ahn[1,2*]

[1]Cell factory Research Centre, Korea Research Institute of Bioscience & Biotechnology, 125 Gwahak-ro, Yuseong-gu, Daejeon 34141, Republic of Korea

[2]Department of Environmental Biotechnology, KRIBB School of Biotechnology, University of Science and Technology, Daejeon 34113, Republic of Korea

*Corresponding author

Chi-Yong Ahn

E-mail: cyahn@kribb.re.kr

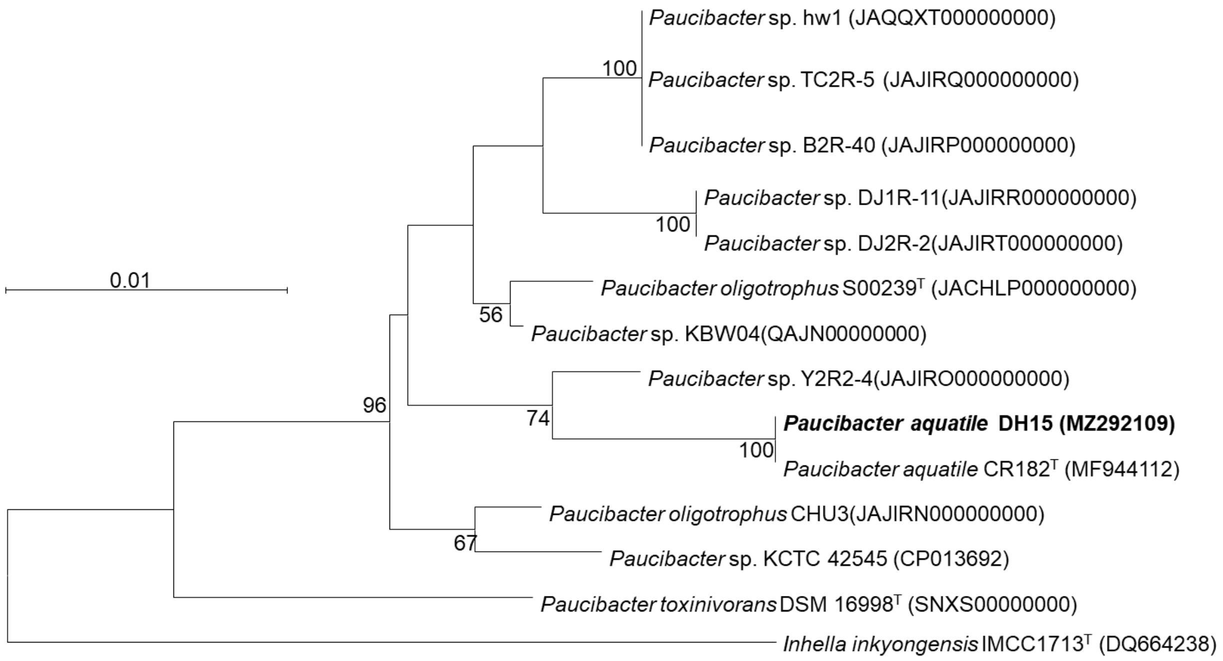

Fig. S1. Neighbor-joining phylogenetic tree based on the 16S rRNA gene sequences depicting the position of strain DH15 among the members of the genus *Paucibacter*. Bootstrap values (≥ 50%) based on 1000 replications are shown at the nodes.

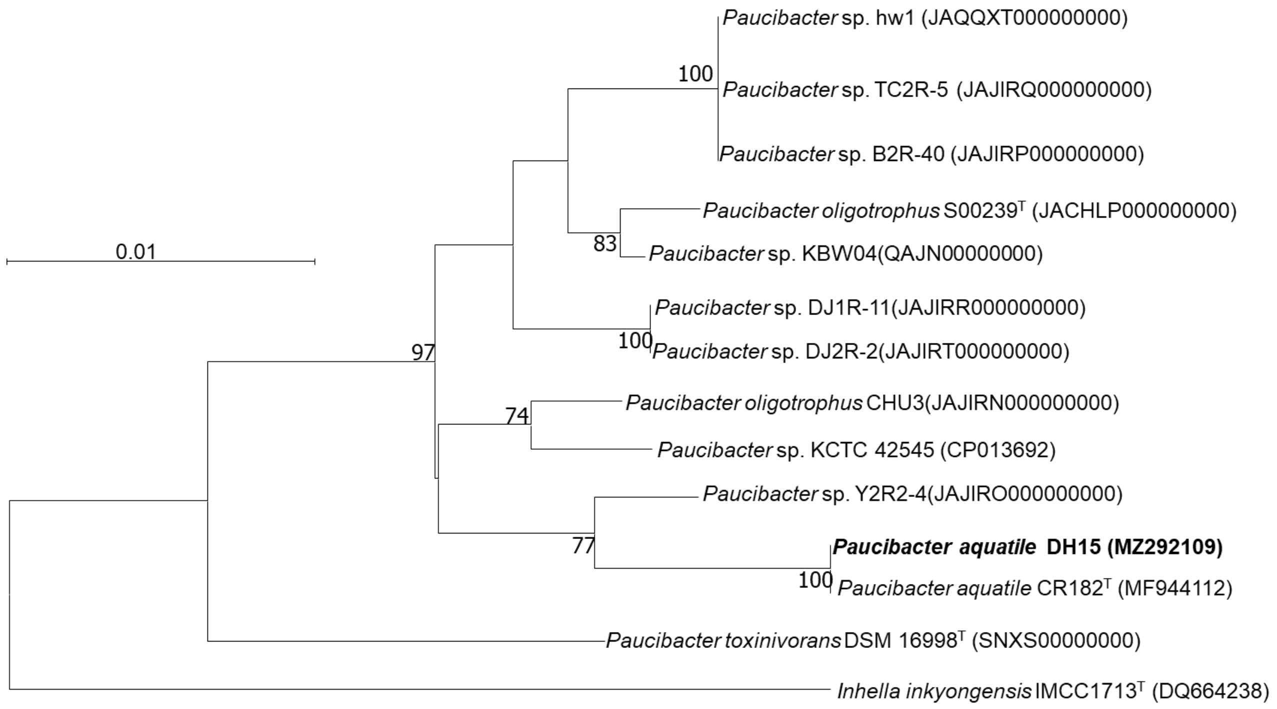

Fig. S2. Minimum-evolution phylogenetic tree based on the 16S rRNA gene sequences depicting the position of strain DH15 among the members of the genus *Paucibacter*. Bootstrap values (≥ 50%) based on 1000 replications are shown at the nodes.

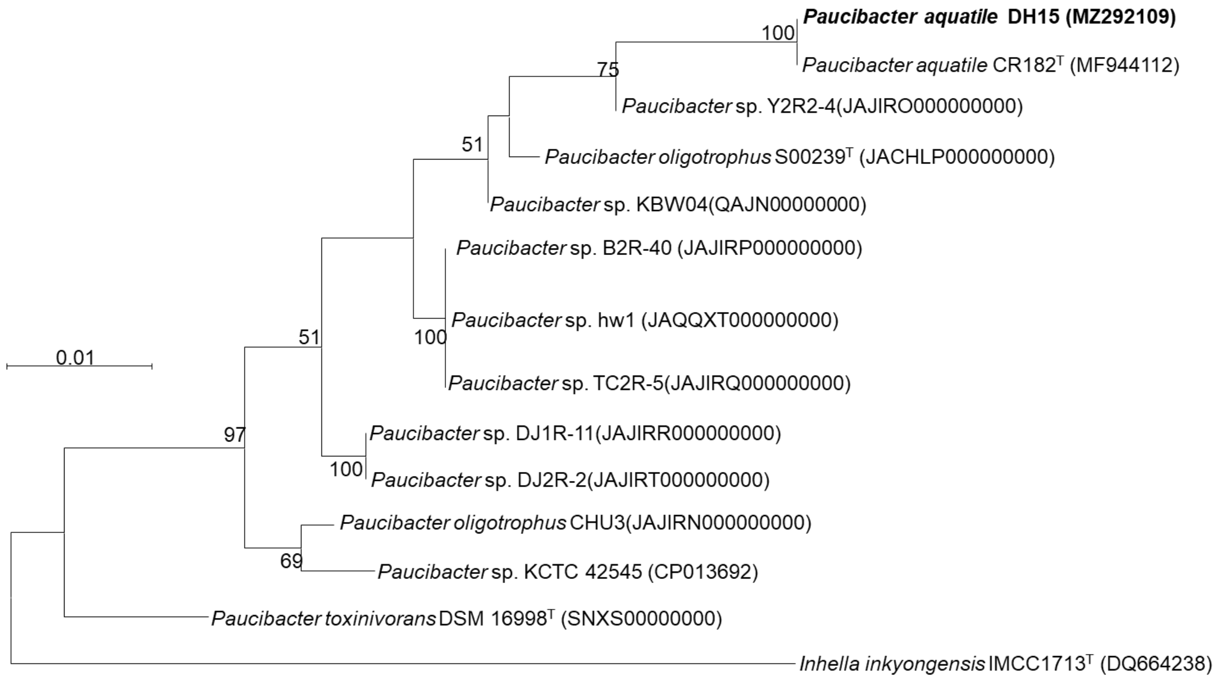

Fig. S3. Maximum-likelihood phylogenetic tree based on the 16S rRNA gene sequences depicting the position of strain DH15 among the members of the genus *Paucibacter*. Bootstrap values (≥ 50%) based on 1000 replications are shown at the nodes.

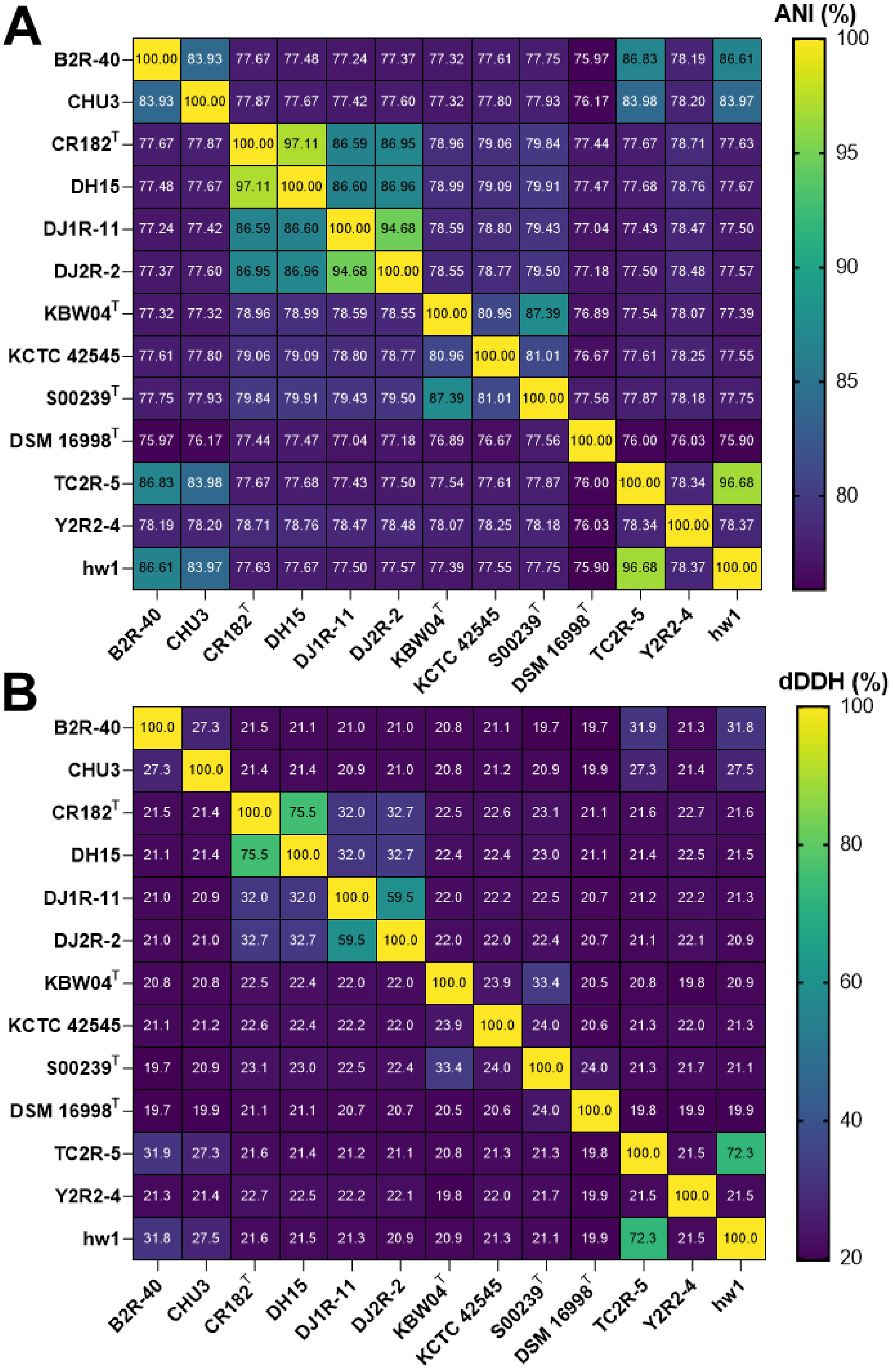

Fig. S4. Heatmaps of average nucleotide identity (ANI) (A) and digital DNA-DNA hybridization (dDDH) (B) values amongst different strains of the genus *Paucibacter*.

Table S1. Descriptions of modules found in the genomes of *Paucibacter* species using BlastKOALA.

Functional category	Pathway modules	Module	Description
Carbohydrate metabolism	Central carbohydrate metabolism	M00001	Glycolysis (Embden-Meyerhof pathway), glucose => pyruvate
		M00002	Glycolysis, core module involving three-carbon compounds
		M00003	Gluconeogenesis, oxaloacetate => fructose-6P
		M00307	Pyruvate oxidation, pyruvate => acetyl-CoA
		M00009	Citrate cycle (TCA cycle, Krebs cycle)
		M00010	Citrate cycle, first carbon oxidation, oxaloacetate => 2-oxoglutarate
		M00011	Citrate cycle, second carbon oxidation, 2-oxoglutarate => oxaloacetate
		M00007	Pentose phosphate pathway, non-oxidative phase, fructose 6P => ribose 5P
		M00005	PRPP biosynthesis, ribose 5P => PRPP
		M00008	Entner-Doudoroff pathway, glucose-6P => glyceraldehyde-3P + pyruvate
	Other carbohydrate metabolism	M00012	Glyoxylate cycle
		M00632	Galactose degradation, Leloir pathway, galactose => alpha-D-glucose-1P
		M00549	Nucleotide sugar biosynthesis, glucose => UDP-glucose
		M00554	Nucleotide sugar biosynthesis, galactose => UDP-galactose
		M00854	Glycogen biosynthesis, glucose-1P => glycogen/starch
		M00855	Glycogen degradation, glycogen => glucose-6P
		M00909	UDP-N-acetyl-D-glucosamine biosynthesis, prokaryotes, glucose => UDP-GlcNAc
		M00012	Glyoxylate cycle

		M00741	Propanoyl-CoA metabolism, propanoyl-CoA => succinyl-CoA
Energy metabolism	Carbon fixation	M00165	Reductive pentose phosphate cycle (Calvin cycle)
		M00166	Reductive pentose phosphate cycle, ribulose-5P => glyceraldehyde-3P
		M00167	Reductive pentose phosphate cycle, glyceraldehyde-3P => ribulose-5P
		M00168	CAM (Crassulacean acid metabolism), dark
		M00169	CAM (Crassulacean acid metabolism), light
		M00579	Phosphate acetyltransferase-acetate kinase pathway, acetyl-CoA => acetate
	Nitrogen metabolism	M00530	Dissimilatory nitrate reduction, nitrate => ammonia
		M00529	Denitrification, nitrate => nitrogen
	Sulfur metabolism	M00595	Thiosulfate oxidation by SOX complex, thiosulfate => sulfate
	Photosynthesis	M00597	Anoxygenic photosystem II
	ATP synthesis	M00144	NADH: quinone oxidoreductase, prokaryotes
		M00149	Succinate dehydrogenase, prokaryotes
		M00151	Cytochrome bc1 complex respiratory unit
		M00155	Cytochrome c oxidase, prokaryotes
		M00153	Cytochrome bd ubiquinol oxidase
		M00417	Cytochrome o ubiquinol oxidase
		M00156	Cytochrome c oxidase, cbb3-type
		M00157	F-type ATPase, prokaryotes and chloroplasts
Lipid metabolism	Fatty acid metabolism	M00082	Fatty acid biosynthesis, initiation
		M00083	Fatty acid biosynthesis, elongation
		M00086	beta-Oxidation, acyl-CoA synthesis
		M00087	beta-Oxidation
	Lipid metabolism	M00093	Phosphatidylethanolamine (PE) biosynthesis, PA => PS => PE

Nucleotide metabolism	Purine metabolism	M00048	De novo purine biosynthesis, PRPP + glutamine => IMP
		M00049	Adenine ribonucleotide biosynthesis, IMP => ADP,ATP
		M00050	Guanine ribonucleotide biosynthesis, IMP => GDP,GTP
		M00053	Deoxyribonucleotide biosynthesis, ADP/GDP/CDP/UDP => dATP/dGTP/dCTP/dUTP
		M00958	Adenine ribonucleotide degradation, AMP => Urate
		M00959	Guanine ribonucleotide degradation, GMP => Urate
	Pyrimidine metabolism	M00052	Pyrimidine ribonucleotide biosynthesis, UMP => UDP/UTP,CDP/CTP
		M00938	Pyrimidine deoxyribonucleotide biosynthesis, UDP => dTTP
		M00046	Pyrimidine degradation, uracil => beta-alanine, thymine => 3-aminoisobutanoate
Amino acid metabolism	Serine and threonine metabolism	M00020	Serine biosynthesis, glycerate-3P => serine
		M00018	Threonine biosynthesis, aspartate => homoserine => threonine
		M00555	Betaine biosynthesis, choline => betaine
		M00621	Glycine cleavage system
	Cysteine and methionine metabolism	M00021	Cysteine biosynthesis, serine => cysteine
		M00035	Methionine degradation
	Branched chain amino acid metabolism	M00432	Leucine biosynthesis, 2-oxoisovalerate => 2-oxoisocaproate
		M00019	Valine/isoleucine biosynthesis, pyruvate => valine / 2-oxobutanoate => isoleucine
		M00570	Isoleucine biosynthesis, threonine => 2-oxobutanoate => isoleucine
		M00036	Leucine degradation, leucine => acetoacetate + acetyl-CoA
	Lysine metabolism	M00016	Lysine biosynthesis, succinyl-DAP pathway, aspartate => lysine
	Arginine and proline metabolism	M00028	Ornithine biosynthesis, glutamate => ornithine
		M00844	Arginine biosynthesis, ornithine => arginine

		M00015	Proline biosynthesis, glutamate => proline
		M00970	Proline degradation, proline => glutamate
	Polyamine biosynthesis	M00133	Polyamine biosynthesis, arginine => agmatine => putrescine => spermidine
	Histidine metabolism	M00045	Histidine degradation, histidine => N-formiminoglutamate => glutamate
	Aromatic amino acid metabolism	M00022	Shikimate pathway, phosphoenolpyruvate + erythrose-4P => chorismate
		M00023	Tryptophan biosynthesis, chorismate => tryptophan
		M00024	Phenylalanine biosynthesis, chorismate => phenylpyruvate => phenylalanine
		M00025	Tyrosine biosynthesis, chorismate => HPP => tyrosine
		M00533	Homoprotocatechuate degradation, homoprotocatechuate => 2-oxohept-3-enedioate
	Other amino acid metabolism	M00118	Glutathione biosynthesis, glutamate => glutathione
		M00948	Hydroxyproline degradation, trans-4-hydroxy-L-proline => 2-oxoglutarate
Glycan metabolism	Lipopolysaccharide metabolism	M00063	CMP-KDO biosynthesis
Metabolism of cofactors and vitamins	Cofactor and vitamin metabolism	M00115	NAD biosynthesis, aspartate => quinolinate => NAD
		M00120	Coenzyme A biosynthesis, pantothenate => CoA
		M00123	Biotin biosynthesis, pimeloyl-ACP/CoA => biotin
		M00881	Lipoic acid biosynthesis, plants and bacteria, octanoyl-ACP => dihydrolipoyl-E2/H
		M00126	Tetrahydrofolate biosynthesis, GTP => THF
		M00880	Molybdenum cofactor biosynthesis, GTP => molybdenum cofactor
		M00140	C1-unit interconversion, prokaryotes

		M00121	Heme biosynthesis, plants and bacteria, glutamate => heme
		M00122	Cobalamin biosynthesis, cobyrinate a,c-diamide => cobalamin
		M00117	Ubiquinone biosynthesis, prokaryotes, chorismate (+ polyprenyl-PP) => ubiquinol
Biosynthesis of terpenoids and polyketides	Polyketide sugar unit biosynthesis	M00793	dTDP-L-rhamnose biosynthesis
Signature modules	Drug resistance	M00627	beta-Lactam resistance
